# The utility of the mannitol challenge in the assessment of chronic cough: a pilot study

**DOI:** 10.1186/1745-9974-4-10

**Published:** 2008-11-18

**Authors:** Amisha Singapuri, Susan McKenna, Christopher E Brightling

**Affiliations:** 1 Institute of Lung Health, University of Leicester, Glenfield Hospital, Groby Road, Leicester, LE3 9QP, UK

## Abstract

There is a need for more objective outcome measures for chronic cough. In this pilot study we sought to investigate the utility of the mannitol challenge as a cough-provocation test in non-asthmatic chronic cough. We studied 16 healthy controls and 13 subjects with chronic cough. We assessed cough severity using a visual analogue score, capsaicin cough sensitivity, health status using the Leicester Cough Questionnaire and the dose of mannitol to cause 2 (C2) or 5 (C5) coughs. In all of the subjects with chronic cough and 6 of the controls we assessed the 1-week repeatability of the mannitol challenge. We found that in those subjects with chronic cough the geometric mean (logSEM) mannitol C2 and C5 was heightened compared to controls (C2: 4 (0.2) versus 16 (0.1); p = 0.04 and C5: 63 (0.1) versus 251 (0.1); p = 0.04). Cough visual analogue score, capsacin-induced cough sensitivity and health status were also altered in chronic cough compared to healthy controls, but in those subjects with chronic cough none of these outcomes was correlated with the mannitol C2 or C5. The repeatability of the mannitol challenge assessed by intraclass correlation was C2 = 0.53 and C5 = 0.59. A cut-off in the dose of mannitol of 62 mg/ml for C2 and 550 mg/ml for C5 had a sensitivity of 69 and 62% and specificity of 69 and 81% respectively to distinguish chronic coughers from healthy controls. In conclusion, the mannitol challenge my have potential as a novel cough challenge test and further work is required to extend our findings and to assess whether it has utility in different causes of chronic cough.

## Findings

Chronic cough is the most common presenting symptom in primary care; is a significant cause of morbidity and a considerable health economic burden [1]. The need for objective outcome measures for cough has lead to the development of cough challenge tests to assess cough sensitivity [2], health status questionnaires [3] and most recently the development of cough monitors [4,5]. However, there remains debate over the clinical utility of these tests. Asthma is one of the commonest causes of cough [6] and therefore bronchial challenge is often included in the clinical investigations of patients with chronic cough. The mannitol challenge is a novel indirect bronchial challenge [7,8], which exerts an osmotic effect on the airway and consequently has the potential to lead to mast cell activation [9]. One of the early observations in the development of the mannitol challenge was that it has a tussive effect as has been reported for methacholine and histamine challenge tests [10]. Indeed asthmatics cough more than controls in response to manntiol and this effect is independent of bronchoconstriction [11]. Therefore the mannitol challenge has the potential to be used both to assess airway hyperrresponsiveness and cough sensitivity. We hypothesised that subjects with non-asthmatic chronic cough also have a heightened cough in response to mannitol and that this test may be a valid outcome measure in chronic cough. To test our hypothesis we examined the number of coughs induced by mannitol during a challenge and assessed the 1-week repeatability of this test in a group of healthy controls and subjects with non-asthmatic chronic cough.

Subjects were recruited from hospital staff or from the respiratory clinics at Glenfield Hospital, Leicester, UK. Chronic cough was defined as a cough > 8 weeks as per American College of Chest Physician Guideline (ACCP) [12]. Healthy volunteers had no respiratory symptoms. All subjects had normal spirometry, were non-smokers and had < 10 pack year history. Ethical approval for the study was given by the Leicestershire, Northamptonshire and Rutland Research Ethics Committee. All subjects gave written informed consent. Subjects were assessed on three occasions prior to the commencement of therapeutic trials for their cough. At visit one, subjects' demographics, quality of life using the Leicester Cough Questionnaire (LCQ) [3], cough severity assessed by the visual analogue score (VAS) [13], spirometry and cough reflex sensitivity with capsaicin cough challenge [2,14,15] was measured. All of the subjects with chronic cough and 6 of the controls attended on two further occasions separated by one week (visits 2 and 3) where spirometry and the mannitol challenge were completed. In brief, mannitol dry powder capsules (Gift from Pharmaxis) were administered in ascending doubling doses ranging from 5 mg to 635 mg via an Osmohaler. The FEV_1 _was measured between each dose of mannitol to measure a drop in lung function. An empty capsule was administered prior to the 5 mg dose, as a placebo. The numbers of coughs within the first 30 seconds following mannitol administration were counted. The challenge was terminated if the subject's FEV_1 _dropped by 15% (PD15) or more or when the highest dose of mannitol had been attained.

All statistical tests were performed using Prism version 4. The concentration of capsaicin or mannitol that caused two coughs (C2) and five coughs (C5) were calculated by the log-dose-response curves. Comparisons between groups were made using t-tests or Mann-Whitney-U test for parametric and non-parametric data respectively. Repeatability was assessed by intra-class correlation and presented as Bland-Altman plots. Receiver-operator curves were generated for mannitol C2 and C5. Correlations were made between the mannitol challenge and other cough outcome measures using spearman rank correlations. A p-value of < 0.05 was considered to be statistically significant.

**Figure 1:**
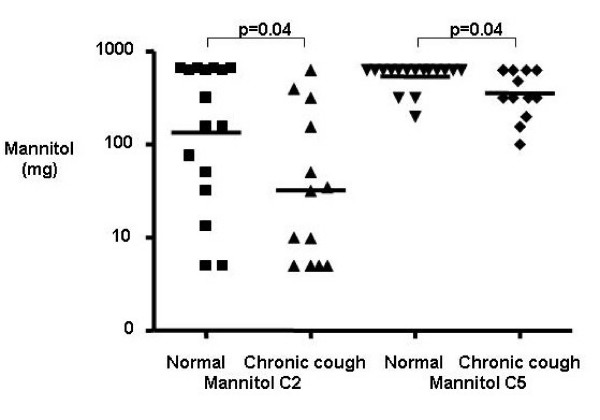
**Heightened mannitol cough sensitivity in chronic cough**. Mannitol C2 and C5 for subjects with chronic cough and healthy controls.

**Figure 2:**
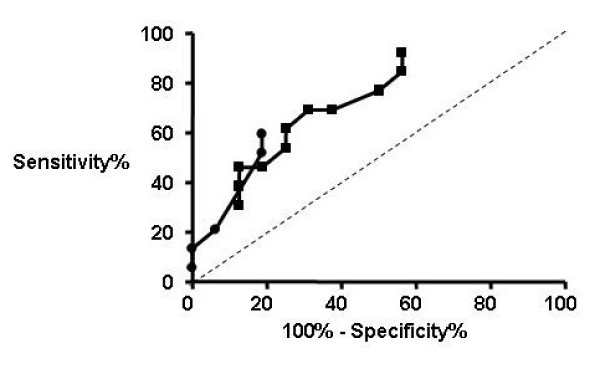
**Receiver-operator curves for mannitol challenge**. The receiver-operator curves for C2 (■) and C5 (●).

**Figure 3:**
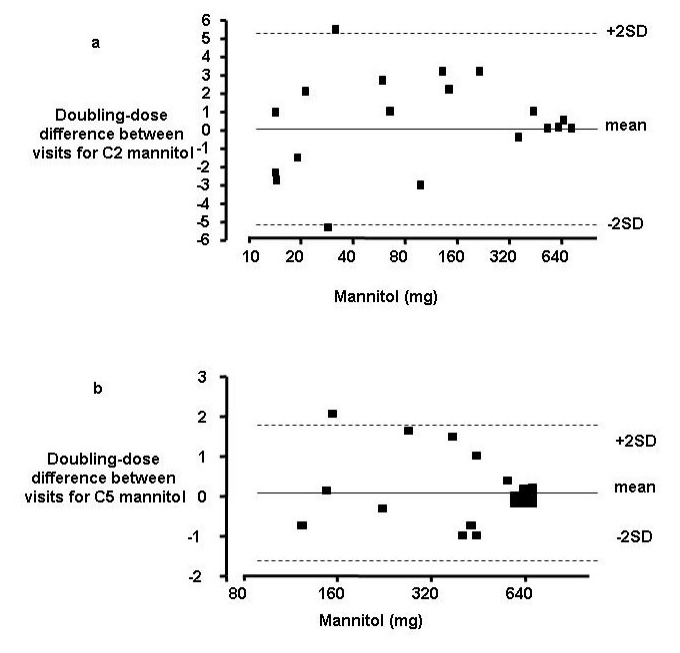
**Repeatability of mannitol challenge**. Bland-Altman plots of the 1-week repeatability of a) the mannitol C2 and b) C5.

Clinical characteristics for subjects are as shown in table 1. Of the 13 subjects with chronic cough the final diagnoses were upper airway cough syndrome 3, gastro-oesophageal reflux 3, unexplained 3, non-asthmatic eosinophilic bronchitis 2, post-infectious 1, chronic bronchitis 1. The subjects with chronic cough had impaired cough-related health status assessed by the LCQ, increased cough VAS and heightened cough sensitivity assessed by capsaicin cough challenge compared to healthy controls. The mannitol C2 and C5 were decreased in subjects with chronic cough compared to healthy controls demonstrating a heightened response to mannitol (Table 1 and Figure 1). None of the subjects had airway hyperresponsiveness in response to mannitol. Receiver-operator curves for mannitol C2 and C5 are as shown Figure 2. The area under the curve was significantly increased for mannitol C2 (mean [95% CI] 0.73 [0.54–0.91]; p = 0.039) and C5 (0.72 (0.52–0.91]; p = 0.049). A cut-off in the dose of mannitol of 62 mg/ml for C2 and 550 mg/ml for C5 had a sensitivity of 69 and 62% and specificity of 69 and 81% respectively (Figure 2). Bland-Altman plots for the within subject repeatability of the mannitol C2 and C5 are as shown (Figure 3a, b). The mean [SD] within subject repeatability for mannitol C2 and C5 was 0.2 doubling-doses [2.6], and 0.13 doubling-doses [0.9] respectively. Therefore to detect a difference of 1 doubling-dose in mannitol C5 13 subjects are required in each group to have 80% power at the 5% level. The intraclass correlation was C2 = 0.53 and C5 = 0.59. In the whole group there were strong correlations between the mannitol C5 and the total LCQ, its domains and capsaicin C5 (Table 2). These correlations did not extend to the subjects with chronic cough alone. Similarly, there were no significant correlations between mannitol C2 and other cough measures in those subjects with chronic cough.

**Table 1:** Clinical characteristics

	**Normal**	**Chronic cough**
**Number**	16	13
**Age^#^**	48 (4)	54 (4)
**Male/Female**	7/9	3/10
**FEV_1_% predicted^#^**	106 (4)	91 (4)*
**FEV_1_/FVC %^#^**	82 (1)	81 (1)
**Total LCQ^#^**	6.9 (0.01)	5.7 (0.33)*
**Physical domain^#^**	6.9 (0.03)	5.5 (0.30)*
**Psychosocial domain^#^**	7.0 (0.0)	6.0 (0.38)*
**Social domain^#^**	7.0 (0.02)	5.8 (0.44)*
**Cough VAS^#^**	0.9 (0.4)	29.5 (6.3)*
**Mannitol PD15**	> 2.8	> 2.8
**Capsaicin C2∧**	16 (0.1)	4 (0.2)*
**Capsaicin C5∧**	251 (0.1)	63 (0.1)*
**Mannitol C2∧**	126 (0.2)	32 (0.2)*
**Mannitol C5∧**	501 (0.04)	316 (0.07)*

#mean (SEM), ∧geometric mean (logSEM), *p < 0.05

**Table 2:** Univariate analysis of the correlations between mannitol C2 and C5 and other cough outcome measures in the whole group and chronic coughers alone

Mannitol Challenge	C2 Whole group		C2 Chronic cough		C5 Whole group		C5 Chronic cough	
Age ^#^	r = -0.2	p = 0.31	r = 0.17	p = 0.59	r = -.35	p = 0.06	r = -0.47	p = 0.1
Total LCQ	r = 0.25	p = 0.19	r = -0.09	p = 0.77	r = 0.47	**p = 0.01**	r = 0.38	p = 0.2
Physical domain	r = 0.23	p = 0.23	r = -0.16	p = 0.6	r = 0.48	**p = 0.01**	r = 0.40	p = 0.18
Psychosocial domain	r = 0.31	p = 0.10	r = 0.12	p = 0.71	r = 0.55	**p = 0.002**	r = 0.48	p = 0.10
Social domain	r = 0.38	**p = 0.04**	r = 0.18	p = 0.57	r = 0.56	**p = 0.002**	r = 0.53	p = 0.07
Cough VAS	r = -0.31	p = 0.10	r = -0.09	p = 0.77	r = -0.38	**p = 0.04**	r = -0.32	p = 0.29
Capsaicin C2	r = 0.19	p = 0.45	r = -0.01	p = 0.96	r = -0.04	P = 0.88	r = -0.37	p = 0.22
Capsaicin C5	r = 0.37	**p = 0.05**	r = 0.05	p = 0.88	r = 0.60	**p = 0.006**	r = -0.35	p = 0.25

In this pilot study, we report for the first time that mannitol-provoked cough is a repeatable test and is increased in non-asthmatic chronic cough. The advantages of the mannitol challenge compared to more established cough challenge tests is that administration is simple, whereas other tests often require preparation of solutions needing laboratory support, and the mannitol challenge provides additional information on AHR. Our findings support our hypothesis that mannitol-provoked cough is increased in non-asthmatic chronic cough as has been reported in asthma [11]. However, there was considerable overlap in the mannitol C2 and C5 between subjects with cough and controls as has been previously observed with other cough challenge tests [14]. Although, the ROC area under the curve for mannitol C2 and C5 showed that the accuracy of the test was good, the sensitivity and specificity of the test was only fair questioning the predictive value of the test and therefore its utility. The short-term repeatability was reasonable, but was not as good as previously reported for the capsaicin challenge [14]. This pilot study therefore suggests that further work is warranted to assess whether the mannitol challenge has a place as a cough outcome measure.

The cause of mannitol-provoked cough is unknown. It is likely that it mediates its effect indirectly via activation of mast cells in the superficial airway to release mediators, which in turn activate local cough receptors. An earlier study in asthma demonstrated that the degree of bronchoconstriction in response to mannitol and the cough response are independent [11]. This supports the view that mast cell localisation to the epithelium and airway smooth muscle are features of asthma, but may co-exist to different degrees in the same individual [16,17]. Asthmatics with increased mast cell number in the airway smooth muscle bundle have heightened AHR [15] and it is possible that the number and state of activation of mast cells in the epithelium determines the cough response. Future studies to investigate the mechanisms of mannitol-provoked cough and its relationship to the airway immunopathology are required.

There a number of potential shortcomings of our study. This was a pilot study of a small number of subjects with non-asthmatic chronic cough. We are therefore unable to determine whether mannitol-provoked cough has a strong association with different causes of cough. Its utility as a cough outcome may therefore differ dependent upon the aetiology of the cough. We have only assessed the short-term repeatability. To fully address the clinical utility of mannitol in the assessment of cough future work will need to determine repeatability over a longer period and investigate responsiveness to therapy in more subjects.

In conclusion, the mannitol challenge may have potential as a novel cough challenge test. However, its repeatability and ability to discriminate between subjects with non-asthmatic chronic cough in this pilot study was not yet sufficient to recommend its routine use. Our findings need to be replicated in a larger series of chronic coughers and in particular it may be informative to examine response in chronic coughers with different aetiologies.

## Competing interests

The authors declare that they have no competing interests.

## Authors' contributions

AS and SM undertook the clinical assessments. AS and CB prepared the manuscript. All authors have read and approved the manuscript. CB supervised the study.
